# Cause and Treatment of Rheumatism

**Published:** 1904-09

**Authors:** 


### CAUSE AND TREATMENT OF RHEUMATISM.

W. H. Porter (*Post-Graduate*) thinks that rheumatism is caused by overfeeding. In some cases it is the result of eating too much vegetable food; in other cases it is due to eating too much meat. In either case it is the result of deficient oxygenation. The essential thing, therefore, in treatment is to regulate the diet—to keep the patient well within the oxidizing capacity of his system. For immediate relief salicylic acid is, of course, the indicated remedy. Dr. Porter likes the following formula:

| | | |
|---|---|---|
| ℞ | Acid salicylic | dr. iii. |
| | Sodium bicarbonate | dr. ii. |
| | Potassium iodid | dr. i.—iii. |
| | Elix. of gaultheria | oz. i. |
| | Glycerin | oz. i. |
| | Aq | oz. iv. |

M. Sig.—One tablespoonful every two hours.—*The Medical Standard.*

